# CLIP-Mono3D: End-to-End Open-Vocabulary Monocular 3D Object Detection via Semantic–Geometric Similarity

**DOI:** 10.3390/s26082380

**Published:** 2026-04-13

**Authors:** Zichong Gu, Shiyi Mu, Hanqi Lyu, Shugong Xu

**Affiliations:** 1 School of Communication and Information Engineering, Shanghai University, Shanghai 200444, China; guzichong123@shu.edu.cn (Z.G.); shiyimu@shu.edu.cn (S.M.); lvhanqi@shu.edu.cn (H.L.); 2 Department of Intelligent Science, Xi’an Jiaotong-Liverpool University, Suzhou 215123, China

**Keywords:** 3D monocular object detection, open-vocabulary learning, autonomous driving

## Abstract

Open-vocabulary 3D object detection (OV-3DOD) is crucial for real-world perception, yet existing monocular methods are often limited by predefined categories or heavy reliance on external 2D detectors. In this paper, we propose CLIP-Mono3D, an end-to-end one-stage transformer framework that directly integrates vision–language semantics into monocular 3D detection. By leveraging CLIP-derived semantic priors and grounding object queries in semantically salient regions, our model achieves robust zero-shot generalization to novel categories without requiring auxiliary 2D detectors. Furthermore, we introduce OV-KITTI, a large-scale benchmark extending KITTI with 40 new categories and over 7000 annotated 3D bounding boxes. Extensive experiments on OV-KITTI, KITTI, and Argoverse demonstrate that CLIP-Mono3D achieves competitive performance in open-vocabulary scenarios.

## 1. Introduction

Monocular 3D object detection estimates the 3D bounding boxes of objects from a single RGB image, an approach that offers compelling cost and deployment advantages over LiDAR-based or multi-sensor fusion systems. This capability is particularly valuable in large-scale applications such as autonomous driving, robotics, and augmented reality, where hardware simplicity and scalability are paramount. However, despite its practical appeal, monocular 3D detection remains significantly more challenging than its 2D counterpart. This difficulty stems primarily from the inherently ill-posed nature of depth recovery from a single viewpoint and the scarcity of large-scale, semantically diverse 3D annotations.

Recent years have witnessed substantial progress driven by benchmark datasets such as KITTI [1], SUN RGB-D [2], ScanNet V2 [3], and nuScenes [4]. Yet, as summarized in Table 1, these datasets are largely constrained in semantic scope, typically encompassing only nine to 23 object categories. Most of these categories are restricted to traffic-related agents, such as cars and pedestrians, or common indoor items. In contrast, modern 2D detection benchmarks like COCO and Objects365 cover hundreds of categories, enabling robust generalization across diverse visual concepts. This semantic gap severely limits the applicability of current 3D detectors in open-world environments, where systems must recognize and localize objects beyond a fixed, predefined set.

**Figure 1:**
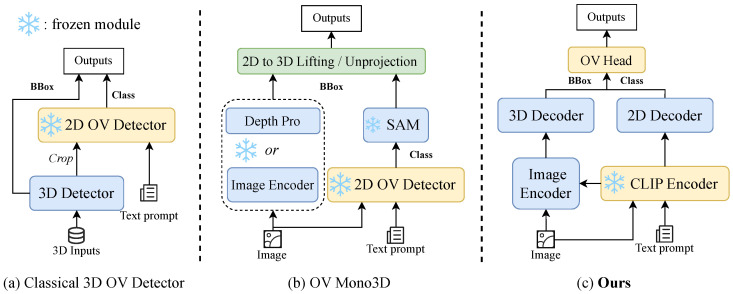
Comparison with existing open-vocabulary 3D detectors. (**a**) Traditional paradigm using frozen 2D detectors. (**b**) Monocular extensions like OVMono3D. (**c**) Our end-to-end CLIP-Mono3D.

To bridge this gap, open-vocabulary 3D object detection has emerged as a promising research direction. The goal is to enable models to detect and localize objects described by arbitrary natural language prompts, including those unseen during training. Existing approaches, however, predominantly follow a two-stage paradigm. They first leverage a pre-trained 2D open-vocabulary detector to extract semantic proposals, which are then fed into a class-agnostic 3D detector for geometric regression, as illustrated in Figure 1a. While effective, this design introduces several practical limitations: (i) it requires heavy reliance on external 2D detectors and their associated supervision; (ii) it involves multi-stage training pipelines that are difficult to optimize jointly; and (iii) many methods still depend on point cloud priors or depth estimators, undermining the simplicity of the monocular setting.

Recent attempts, such as OVMono3D [5], have sought to mitigate these issues by incorporating foundation models like SAM for segmentation priors or monocular depth estimators. Nevertheless, they remain fundamentally dependent on pre-trained 2D detectors and auxiliary data sources, preventing true end-to-end training from 3D supervision alone.

Despite these efforts, a critical research gap remains: achieving true end-to-end open-vocabulary 3D detection solely from monocular images, without the architectural complexity and inference latency introduced by auxiliary 2D detectors, multi-stage pipelines, or external depth priors.

In this work, we present CLIP-Mono3D, a novel, end-to-end trainable framework for open-vocabulary monocular 3D object detection. Built upon the MonoDGP architecture [6], our method integrates semantic knowledge directly into the 3D detection pipeline by leveraging a pre-trained FG-CLIP visual–language encoder [7]. Unlike prior approaches, CLIP-Mono3D eliminates the need for an external 2D detector by fusing CLIP-derived visual–semantic features with geometric representations via cross-modal attention. By initializing detection queries using language embeddings, our model achieves zero-shot generalization to novel categories without additional 2D supervision, representing a significant step toward practical and generalizable monocular 3D perception.

To facilitate research in this under-explored direction, we further introduce OV-KITTI, a new benchmark that extends the original KITTI dataset with 40 additional object categories, including animals and everyday items. As shown in Table 1, OV-KITTI not only expands semantic coverage but also provides more diverse shape and scale priors, which help alleviate the depth ambiguity inherent in monocular setups. The dataset is carefully curated to ensure balanced distributions between base and novel categories, enabling fair evaluation of open-vocabulary generalization.

The significance of our work lies in three key contributions:

1. We propose CLIP-Mono3D, an end-to-end framework that unifies semantic and geometric reasoning. By introducing a cross-modal semantic–geometric fusion module, we inject fine-grained semantic clues into geometric features via a lightweight residual connection, enhancing semantic awareness without disrupting pre-trained geometric cues.
2. We design a novel query initialization strategy that converts 2D semantic probability maps into explicit 3D query positions. This mechanism significantly improves 3D center localization and recall for open-vocabulary objects compared to standard learned queries.
3. We introduce OV-KITTI, a large-scale benchmark with controlled semantic and size distributions. Extensive experiments on OV-KITTI, KITTI, and Argoverse demonstrate that CLIP-Mono3D achieves competitive performance in both closed- and open-vocabulary settings, paving the way for deployment in truly open-world scenarios.

The remainder of this paper is organized as follows. Section 2 reviews related work in monocular and open-vocabulary 3D object detection. Section 3 details the methodology of the proposed CLIP-Mono3D framework. Section 4 introduces the newly curated OV-KITTI benchmark. Section 5 presents the experimental results and discussions. Finally, Section 6 provides the conclusion of our work.

## 2. Related Work

In this section, we review the recent work highly relevant to our research.

### 2.1. Monocular 3D Object Detection

Monocular 3D object detection localizes 3D bounding boxes from single RGB images, but its development lags behind point cloud and multi-view methods due to ill-posed depth estimation. Current approaches include geometry-based methods and depth-assisted techniques. Geometry-based methods leverage geometric consistency: some reduce the task to 2D detection with 3D–2D projection for center calculation [8,9], while others directly regress 3D boxes via keypoints [10,11]. Enhanced variants incorporate geometric priors like ground planes [12], shape awareness [13], uncertainty modeling [14], and height cues [15]. To address depth perception limitations, depth-assisted methods utilize pseudo-LiDAR representations [16,17], LiDAR-supervised depth estimation [18], frustum discretization for BEV mapping [19], and depth pre-training [20]. Transformer-based designs further integrate visual/depth features [21] and employ depth-guided decoding [22]. Additionally, recent advancements such as MonoCLUE [23] have explored the integration of visual foundation models, utilizing SAM to provide segmentation priors. However, these methods typically operate strictly within a closed-vocabulary setting.

### 2.2. Open-Vocabulary Object Detection

Open-vocabulary object detection handles both seen and unseen categories to accommodate real-world diversity. With CLIP’s rise, 2D open-vocabulary detection has advanced through knowledge distillation from image–text pairs [24], pseudo-label generation [25,26], pre-trained encoders (CLIP [27,28,29,30,31] or BERT [32,33,34]), and generative modeling [35,36]. Transformer-based methods dominate for their effective integration of text encoders.

### 2.3. Open-Vocabulary 3D Object Detection

Extending to 3D space, this task detects unseen categories with higher complexity. Early works used unsupervised clustering for open-set detection [37]. Recent CLIP-inspired approaches include pseudo-label methods where OV3DET [38] trains class-agnostic detectors with 2D-generated labels then aligns 3D, image and language features. Cross-modal alignment techniques combine 3D geometric and 2D semantic priors without 2D detectors [39], or enrich datasets via cross-domain alignment [40]. Knowledge propagation frameworks cyclically transfer 2D semantic knowledge to 3D and geometric cues to 2D [41]. Point cloud–language alignment further bridges modalities [42,43]. Other recent multi-stage paradigms [5,44] extend open-vocabulary capabilities by heavily relying on pre-trained external 2D detectors or foundation models to extract semantic proposals before performing 3D geometric regression. While effective, they inherit the complexity of multi-stage pipelines and depend on external 2D supervision.

## 3. Method

This section details the proposed CLIP-Mono3D framework. We begin by defining the mathematical background of the task and presenting the overall architecture. We then introduce the cross-modal feature fusion module, the language-aware query initialization strategy, describe the open-vocabulary detection head and formulate the corresponding training objectives.

### 3.1. Preliminaries and Overall Architecture

Fundamentally, monocular 3D object detection is constrained by the geometry of projective transformations. Following Hartley and Zisserman [45], the mapping of a 3D point $X={\left[X,Y,Z,1\right]}^{⊤}$ to a 2D pixel coordinate $x={\left[u,v,1\right]}^{⊤}$ is defined by the projection equation λx=K[R|t]X, where K is the intrinsic camera matrix, [R|t] denotes the extrinsic rotation and translation, and λ represents the depth. Recovering X from x is inherently ill-posed since depth λ is lost during projection. Furthermore, practical imaging systems often suffer from optical aberrations, such as radial/tangential distortion and chromatic aberration. These imperfections perturb the ideal linear projection model, introducing non-linear spatial variations that further complicate precise 3D center and geometry recovery.

Open-vocabulary monocular 3D object detection aims to estimate 3D bounding boxes $D={\left\{O_{i}\right\}}_{i=1}^{N}$ from a single RGB image $I\in N^{H\times W\times 3}$, guided by a set of arbitrary text prompts $T=\{\tau_{1},\ldots ,\tau_{K}\}$. Each object $O_{i}$ is parameterized by its 3D center $\left(x_{i},y_{i},z_{i}\right)$, dimensions $\left(l_{i},w_{i},h_{i}\right)$, orientation $\theta_{i}$, and a semantic similarity vector $s_{i}\in {\left[0,1\right]}^{K}$. The similarity score for each prompt is computed as:

(1) $$s_{i}\left[k\right]=\sigma \left(\phi_{\mathrm{img}}{\left(p_{i}\right)}^{⊤}\phi_{\mathrm{text}}\left(\tau_{k}\right)\right),$$

where σ denotes the sigmoid function, $\phi_{\mathrm{img}}$ and $\phi_{\mathrm{text}}$ represent the image and text encoders (e.g., CLIP), and $p_{i}$ denotes the region-level features associated with the *i*th object. This formulation enables the detection of novel categories by replacing fixed-set classification with open-ended semantic matching.

**Figure 2:**
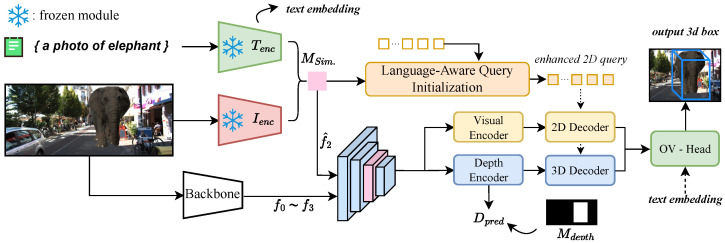
Pipeline of CLIP-Mono3D. Multi-scale visual features are fused with the CLIP similarity matrix ($M_{\mathrm{sim}}$) to form multimodal representations. These representations, along with language-aware queries initialized from $M_{\mathrm{sim}}$, are processed by dual transformer architectures. Depth predictions are supervised via an object-wise depth map ($M_{\mathrm{depth}}$) to output the final open-vocabulary 3D bounding boxes.

As illustrated in Figure 2, CLIP-Mono3D extends the MonoDGP architecture [6]. A ResNet-50 backbone first extracts multi-scale features ${\left\{F_{i}\right\}}_{i=0}^{3}$, which are enhanced by a Region Segmentation Head (RSH) to produce $F_{V}$. These features are fused with CLIP-derived semantics (Section 3.2) and processed by a depth predictor to generate $F_{D}$. Dual transformer encoders independently process these streams, followed by a 2D visual decoder and a 3D depth-guided decoder. To provide explicit geometric priors, language-aware queries (Section 3.3) are initialized from CLIP similarity maps. Final predictions are refined via geometric depth correction and scored against text embeddings.

### 3.2. Cross-Modal Feature Fusion

To bridge the semantic gap between vision and language modalities, we introduce a lightweight cross-modal fusion mechanism that injects text-guided spatial priors into the visual backbone. Given an input image I and prompts T, we extract dense visual features $F_{\mathrm{img}}\in R^{14\times 14\times d}$ and global textual embeddings $F_{\mathrm{txt}}\in R^{K\times d}$ using a frozen CLIP encoder. Freezing CLIP’s parameters is essential to preserve its zero-shot generalization and prevent the loss of rich semantic knowledge during 3D detection training.

Semantically relevant regions are identified by computing a spatial similarity map $S\in R^{14\times 14}$ through the aggregation of cosine similarities across all text tokens:

(2) $$S\left(u,v\right)=\frac{1}{K}\sum_{k=1}^{K}\frac{F_{\mathrm{img}}^{\left(u,v\right)}\cdot F_{\mathrm{txt}}^{\left(k\right)}}{∥F_{\mathrm{img}}^{\left(u,v\right)}∥∥F_{\mathrm{txt}}^{\left(k\right)}∥}.$$

This map functions as a soft attention mask, highlighting regions aligned with the linguistic input. Unlike post hoc filtering methods, our approach integrates this signal early in the feature hierarchy, allowing semantic guidance to inform all downstream components.

The similarity map S is bilinearly upsampled to match the dimensions of the intermediate feature map $F_{V}^{\left(2\right)}\in R^{C\times H_{2}\times W_{2}}$. It is then processed by a convolutional module $\Gamma_{\mathrm{fuse}}$—comprising two 3×3 convolutions with ReLU activations—to refine its spatial structure and match the channel dimensions. The final fused feature map is obtained via an additive residual connection:

(3) $$F_{V}^{\mathrm{enh}(2)}=F_{V}^{\left(2\right)}+\Gamma_{\mathrm{fuse}}\left(\mathrm{Upsample}(S)\right).$$

This residual design ensures that primary geometric and structural information remains intact, which is critical for accurate 3D localization. This early fusion strategy creates a “semantic spotlight” that benefits both the RSH and the subsequent query initialization stage (Section 3.3).

### 3.3. Language-Aware Query Initialization

Standard DETR-like architectures typically initialize object queries as content-agnostic learnable embeddings, which can lead to slow convergence in complex scenes. To address this, we propose a language-aware query initialization strategy that transforms 2D semantic probability maps into explicit 3D geometric anchors.

**Figure 3:**
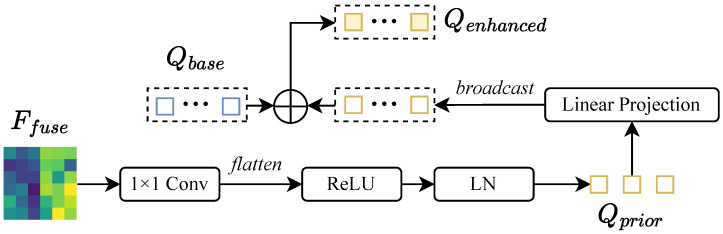
Language-aware query initialization. Top-$N_{q}$ keypoints from the CLIP similarity map are used to sample and aggregate feature priors.

As shown in Figure 3, the initialization involves three steps. First, we interpret the CLIP similarity map S as a spatial probability distribution and identify $N_{q}$ locations $P={\left\{\left(u_{k},v_{k}\right)\right\}}_{k=1}^{N_{q}}$ with the highest activations. These serve as candidate 2D centers for potential 3D objects.

Second, we employ F.grid_sample to sample local descriptors $P_{\mathrm{keypoint}}\in R^{N_{q}\times C}$ from the enhanced feature map $F_{V}^{\mathrm{enh}(2)}$ at coordinates P. This differentiable operation ensures that each query is initialized with features specific to its corresponding object region.

Third, a global semantic prior is distilled from these descriptors via a two-layer MLP:

(4) $$Q_{\mathrm{prior}}=W_{2}\;\delta \left(W_{1}\cdot \frac{1}{N_{q}}\sum_{k=1}^{N_{q}}P_{\mathrm{keypoint}}^{\left(k\right)}\right).$$

The base queries $Q\in R^{N_{q}\times 2d}$ consist of a content component $Q_{c}$ and a positional component $Q_{p}$. We use sine–cosine positional encodings of P to initialize $Q_{p}$, while $Q_{c}$ is enhanced by the global prior:

(5) $$Q_{\mathrm{content}}=Q_{c}+Q_{\mathrm{prior}},\;Q=\left[Q_{\mathrm{content}};Q_{p}\right].$$

By grounding queries in semantically verified regions, we transform the detection process from exhaustive spatial searching to targeted localization. This “semantic priming” accelerates training convergence and reduces attention to background clutter. Importantly, the CLIP-derived prior provides a strong inductive bias for open-world concepts. During inference, if no text is provided, the system reverts to base queries ($Q_{\mathrm{prior}}=0$) to maintain backward compatibility.

### 3.4. Open-Vocabulary Detection Head and Loss

The detection head enables open-vocabulary classification by computing semantic similarity between decoder outputs and projected text embeddings. For each output $h\in R^{d_{h}}$, we project it into the CLIP feature space, $v^{\prime }=\mathrm{norm}\left(W_{v}h\right)$, where $W_{v}\in R^{512\times d_{h}}$, and norm(·) denotes $\ell^{2}$ normalization. Similarly, text embeddings $t_{\mathrm{CLIP}}$ are projected via a learnable matrix $W_{t}$ to obtain $t^{\prime }$.This dual-projection design mitigates the domain gap between the detector’s internal representations and CLIP’s pre-trained embeddings.

The similarity score is computed as a temperature-scaled dot product, $s=\tau \cdot v^{\prime ⊤}t^{\prime }$, where the learnable temperature τ=exp(γ) controls the distribution concentration. To handle background regions, we introduce a learnable background embedding $t_{bg}$ appended to the text embeddings.

Training follows a bipartite matching strategy using the Hungarian algorithm. The matching cost incorporates both geometric and semantic terms:

(6) $$C_{\mathrm{match}}=\lambda_{g}\left(∥\Delta c_{3d}{∥}_{1}+{∥\Delta b_{2d}∥}_{1}+\left(1-\mathrm{GIoU}\right)\right)-\lambda_{s}s.$$

Integrating the similarity score *s* into the matching process ensures that predictions are assigned based on both spatial accuracy and semantic coherence, significantly enhancing generalization to unseen classes.

For matched pairs M, we apply a contrastive loss:

(7) $$L_{\mathrm{contrast}}=-\frac{1}{\left|M\right|}\sum_{(i,j)\in M}\log\frac{\exp(s_{ij}/\tau )}{\sum_{k}\exp\left(s_{ik}/\tau \right)}.$$

The total objective combines this with geometric regression losses $L_{g}$:

(8) $$L_{\mathrm{total}}=\lambda_{c}L_{\mathrm{contrast}}+\sum \lambda_{g}L_{g}.$$

To better understand the optimization dynamics, we explicitly formulate the error backpropagation for the contrastive loss. Let $p_{ik}=\frac{\exp(s_{ik}/\tau )}{\sum_{m}\exp\left(s_{im}/\tau \right)}$ denote the predicted softmax probability for class *k*. The gradient of $L_{\mathrm{contrast}}$ with respect to the similarity score $s_{ij}$ for a matched pair (i,j) is given by:

(9) $$\frac{\partial L_{\mathrm{contrast}}}{\partial s_{ij}}=\frac{1}{|M|\tau }\left(p_{ij}-1\right),\;\mathrm{and}\;\mathrm{for}\;k\neq j:\frac{\partial L_{\mathrm{contrast}}}{\partial s_{ik}}=\frac{1}{|M|\tau }p_{ik}.$$

This gradient is strictly bounded within [−1/τ,1/τ]. Since the geometric regression losses ($L_{g}$) employ smooth variants, which also exhibit bounded derivatives, the overall parameter gradients remain stable. This bounded error propagation naturally prevents gradient explosion, demonstrating the theoretical convergence stability of our end-to-end training process.

This co-design ensures that gradients from the contrastive loss are applied to the most semantically relevant predictions, creating a robust optimization cycle that yields a model both geometrically precise and semantically aware.

## 4. OV-KITTI Benchmark

**Figure 4:**
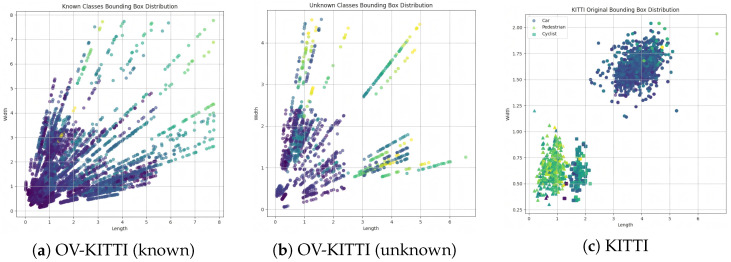
Comparison of bbox size distribution across datasets. The axes represent dimensions of 3D boxes, with brighter colors corresponding to greater heights. OV-KITTI exhibits a more balanced size distribution between known and unknown categories compared to KITTI.

Most existing monocular 3D object detection models rely on the KITTI dataset for training and evaluation. However, this dataset presents several critical limitations. KITTI contains only nine object categories, and prior works predominantly evaluated models on the “Car” category due to the scarcity of other instances. In open-vocabulary or zero-shot learning settings, a common practice involves training on “Car” and “Cyclist” classes while attempting to transfer detection capabilities to the “Pedestrian” category. Nevertheless, as illustrated in Figure 4c, the 3D bounding-box dimensions of the “Car” category differ significantly from those of “Pedestrian” and “Cyclist”. This discrepancy leads to an inherent bias where the detection of “Pedestrian” largely relies on knowledge spillover from “Cyclist” rather than benefiting from the rich feature learning associated with the dominant “Car” category. We argue that this imbalance in both category support and scale distribution within KITTI hinders the development of generalizable 3D detection models.

To overcome these limitations, we introduce OV-KITTI, an augmented benchmark based on the KITTI dataset designed specifically for open-vocabulary monocular 3D detection. We enrich the original dataset with 40 additional object categories sourced from Objaverse [46]. These categories encompass a diverse set of animals and household items. The new objects are carefully selected to avoid semantic overlap with existing traffic participants in KITTI, such as cars and pedestrians, thereby enabling precise supervision and evaluation.

### 4.1. Dataset Construction

The construction of OV-KITTI followed a systematic multi-step pipeline:

(1) Bounding-Box Design: For each new category, we defined physically plausible size constraints for its 3D bounding box. During rendering, the actual box dimensions were randomly sampled within this predefined range to ensure variability. (2) Scene Integration: Using Blender, we rendered 3D meshes into real driving scenes from KITTI. Objects were placed on the ground plane with random rotation, scaling, and translation. We explicitly enforced constraints to ensure no physical overlap with existing objects in the scene. (3) Stereo Rendering: We generated stereo-consistent left and right views for each modified scene. Annotations are provided in the standard KITTI format to ensure seamless compatibility with existing detection frameworks. (4) Balance Control: Special care was taken to balance the 3D box size distributions between known and unknown classes, as shown in Figure 4a,b. This step reduces potential size-related biases and supports a fair evaluation of model generalization capabilities.

### 4.2. Category Statistics

**Figure 5:**
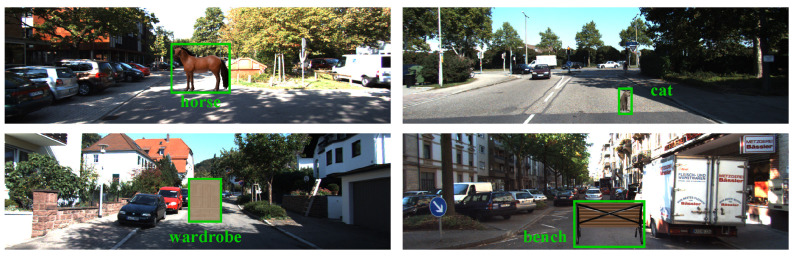
Examples of OV-KITTI. Diverse objects such as animals and household items are rendered with random scales and positions on real KITTI scenes.

We divided the 40 new classes into known (training) and unknown (testing) categories. The known set comprised 32 classes, consisting of 19 household items and 13 animals, while the unknown set contained 8 classes, including 5 items and 3 animals, for zero-shot evaluation. This split ensured diversity and scale balance across training and testing phases. The full category list and instance counts are provided in Table 2. As shown in Figure 5, OV-KITTI contains high-quality renderings of diverse objects under varied scales and contexts. This provides a challenging yet realistic testbed for evaluating open-vocabulary 3D detection performance.

## 5. Experiments

In this section, we comprehensively evaluate the proposed CLIP-Mono3D framework. We first describe the experimental setup, including datasets, evaluation metrics, and implementation details. We then present quantitative comparisons with state-of-the-art methods in both closed-vocabulary and open-vocabulary settings. Furthermore, we provide detailed ablation studies to validate our core components, followed by qualitative visualizations and verifications in unconstrained real-world scenarios.

### 5.1. Experimental Setup

#### 5.1.1. Datasets and Metrics

We evaluated on KITTI and OV-KITTI. For KITTI, we used standard splits (3712 train, 3769 val). For OV-KITTI, we used 5936 training samples (32 classes) and 1545 test samples (8 classes). Original categories were excluded from the open-vocabulary evaluation. We used AP at 40 recall as the metric: $AP_{3D}$ and $AP_{BEV}$ for closed-vocabulary; $AP_{15}$ per category for open-vocabulary.

#### 5.1.2. Settings

We followed MonoDETR-style augmentation and FG-CLIP image preprocessing. The model was implemented in PyTorch 1.13.1 and trained on a single NVIDIA RTX 4090D GPU for 200 epochs with a batch size of eight. We utilized the AdamW optimizer with an initial learning rate of $2\times 10^{-4}$ and a weight decay of $1\times 10^{-4}$. A step learning rate scheduler was employed, decaying the learning rate by a factor of 0.5 at epochs 85, 125, 165.

Regarding hyperparameter configurations, we adopted the validated settings from MonoDGP [6] for geometric components to maintain stable 3D localization. Specifically, the bipartite matching costs for the 3D center, 2D bounding box, and GIoU were set to 10, five, and two, respectively. The corresponding loss coefficients ($\lambda_{g}$) were assigned the same weights. For our open-vocabulary modules, the semantic matching cost ($\lambda_{s}$) and the contrastive loss coefficient ($\lambda_{c}$) were both set to two, with a focal loss α of 0.25. This balance ensured that semantic learning proceeded without destabilizing the established geometric precision during early training stages.

### 5.2. Closed-Vocabulary Results

As shown in Table 3, our method achieves state-of-the-art performance in the standard monocular 3D object detection task. On the KITTI val set, our approach attains 31.40% $AP_{3D}$ under the “easy” difficulty level, surpassing previous methods by a clear margin: +2.84 percentage points over MonoDETR [22] and +1.72 over MonoDGP [6]. The improvements are consistent across moderate and hard difficulty levels, demonstrating enhanced robustness in detecting occluded and distant objects. In BEV detection, our model also sets a new benchmark with 39.47% $AP_{BEV}$ (easy), outperforming both baselines by over 1.5 points.

These gains can be attributed to our improved 2D feature representation enriched with CLIP-derived semantics, which provides stronger cues for depth estimation and spatial localization. Unlike purely geometric reasoning methods, our integration of semantic context allows the detector to better disambiguate scale and position, especially in low-texture or cluttered scenes.

On the OV-KITTI dataset, while the focus shifts toward open-vocabulary generalization, we still report strong closed-vocabulary performance. Our $AP_{25}$ reaches 14.44%, exceeding MonoDGP by 1.11%, which indicates that our enhancements do not compromise detection accuracy on seen classes. This balance between specialization and generalization is crucial for real-world deployment, where systems must handle both known and emerging object categories.

As shown in Table 4, we compare the efficiency metrics with baseline methods. It is important to note that while our framework incorporates a frozen CLIP image encoder of approximately 150 M parameters to extract semantic features, these parameters do not require gradient updates. Consequently, our trainable parameter count of 43.48 M is nearly identical to that of MonoDGP at 43.33 M. This minimal increase of approximately 0.15 M confirms that the performance gains stem from our effective semantic–geometric alignment design rather than a brute-force increase in model capacity. Although the integration of the visual encoder introduces a marginal latency increase, the inference speed of 51 ms per frame remains sufficient for real-time autonomous driving applications.

### 5.3. Open-Vocabulary Results

The true strength of our method lies in its ability to detect objects from previously unseen categories, a key challenge in open-vocabulary 3D detection. As shown in Table 5, our method achieves an overall 5.81% AP on the eight unseen categories in OV-KITTI, outperforming the fine-tuned variant OVMono3D ^†^ by +1.21%.

A critical observation is that fine-tuning on the known training set provides substantial gains for baseline methods, particularly for geometrically complex categories. As shown in the “Gain from FT” rows, OVMono3D ^†^ improves by a remarkable +7.02 points on “tiger” and +1.61 on “giraffe” compared to its non-fine-tuned version. This confirms that exposure to 3D shape priors during fine-tuning is essential for accurate localization of categories largely absent from standard 3D datasets.

Despite this significant boost from fine-tuning, our method still achieves superior performance across most categories. We outperform OVMono3D ^†^ by +2.73 on “tiger” and +1.04 on “giraffe”, demonstrating that our direct integration of vision-language semantics enables better generalization to novel biological shapes, without relying on cascaded 2D detectors or fine-tuning heuristics. For smaller or less frequent objects like “bucket” and “dog”, our method also shows consistent improvements.

The only exception is “wardrobe” where OVMono3D ^†^ retains a slight edge. We attribute this to its stronger pre-trained knowledge of household items and the relatively simple geometric structure of wardrobes, which are more easily captured by existing 2D open-vocabulary detectors. Overall, our results validate that grounding 3D detection directly in language semantics, rather than relying on fine-tuned 2D detectors, leads to superior generalization, especially for structurally diverse and underrepresented categories like animals.

### 5.4. Visualization of Cross-Modal Alignment

**Figure 6:**
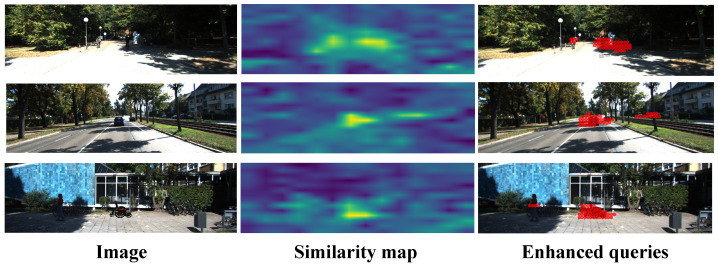
Visualization of similarity map and enhanced queries. The label prompt of each row for input images (**left**) corresponds to KITTI classes: “car” and “wheelchair”. The similarity maps (**middle**) highlights regions matching the text description, and the top-$N_{q}$ keypoints (**right**) visualized in red are used to initialize language-aware object queries.

To elucidate how language priors guide the detection process, we visualize the CLIP-based similarity maps S and their influence on query initialization in Figure 6. As shown in the middle column, S effectively serves as a semantic attention mechanism, highlighting regions aligned with class-specific prompts such as “car” and “wheelchair”. Notably, for the rare class “wheelchair”, which lacks 3D annotations during training, the model identifies plausible candidates by leveraging semantic cues (e.g., human silhouettes with wheels), demonstrating robust open-vocabulary generalization.

The right column illustrates the top-$N_{q}$ keypoints selected for query initialization. These points concentrate on the object’s spatial extent, confirming that our language-aware sampling effectively localizes semantically meaningful regions. These keypoints provide informed feature priors that prime the object queries, enabling the decoder to focus on relevant content from the initial stages.

This visualization validates two key advantages: (1) the similarity map bridges language semantics and visual geometry for zero-shot grounding; (2) language-aware initialization mitigates monocular depth ambiguity by providing semantically grounded spatial anchors.

**Figure 7:**
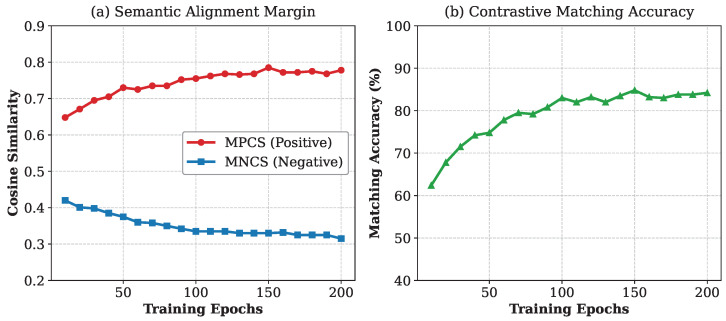
Evolution of cross-modal semantic alignment metrics during training.

To quantitatively validate the effectiveness of our cross-modal feature fusion, we tracked the evolution of semantic alignment metrics during the training process on the validation set. Figure 7 plots the Mean Positive Cosine Similarity (MPCS), the Mean Negative Cosine Similarity (MNCS), and the Contrastive Matching Accuracy across 200 training epochs.

We observe that the network tends to predict relatively high similarity scores across all queries, which is a common characteristic in dense visual–language matching. However, as training progresses, the model successfully learns to discriminate between target and background regions. As shown in Figure 7a, the MPCS steadily increases to approximately 0.78, establishing a clear and robust discriminative margin against the MNCS. Driven by this semantic margin, the contrastive matching accuracy on the assigned positive queries (Figure 7b) rises consistently, converging to around 84.5%. This quantitative trend complements our qualitative similarity maps, confirming that our framework effectively transforms spatial queries into language-aware object representations despite the inherent difficulty of open-vocabulary reasoning.

### 5.5. Ablation Studies

To evaluate the contribution of each proposed component, we conducted comprehensive ablation studies on the OV-KITTI benchmark. Unless otherwise specified, performance was measured by $AP_{25}$ for seen classes (CV) and $AP_{15}$ for unseen categories (OV).

Core Components and Query Initialization. As summarized in Table 6, each module consistently improves performance. While feature fusion enhances the base representation, the language-aware query initialization provides the most significant gain for open-vocabulary generalization (+1.12% OV AP). This suggests that grounding queries in semantic regions from the early decoding stages is more effective for novel objects than relying on generic learnable embeddings.

Regarding the specific initialization design in Table 7, we found that using 50 queries with a global prior aggregated via mean-pooling yielded optimal results. This global context informed each query of the overall semantic landscape, preventing them from over-focusing on isolated, potentially noisy local regions. In contrast, max-pooling over-emphasized the single most salient activation, leading to poorer generalization for diverse scenes. We also compared VLM backbones: FG-CLIP (Frozen) achieved the highest accuracy, while fine-tuning it via LoRA [50] led to a performance drop. This indicates that the 3D dataset’s scale is insufficient to update the VLM without causing catastrophic forgetting of its pre-trained open-world semantic knowledge.

Feature Fusion Strategy and Domain Robustness. Table 8 explores fusion architectures. Additive residual connections at *res2* outperform simple concatenation. This additive design acts as a calibrated semantic spotlight that preserves the geometric integrity of intermediate features, whereas concatenation may introduce noise that disrupts sensitive 3D regressions. Injecting semantic guidance early in the feature hierarchy (stage 2) is crucial for informing downstream depth estimation and query sampling.

To investigate whether the model overfit to synthetic artifacts, we conducted a data-mixing study and present the results in Table 9. We progressively introduced real-world KITTI objects into the training pipeline. Crucially, to ensure a fair comparison, the Seen AP was evaluated exclusively on the original synthetic classes. The results show that adding real-world instances with distinct lighting and textures leads to negligible fluctuations in Unseen AP. This stability confirms that CLIP-Mono3D learns generalized semantic concepts rather than low-level texture priors. The drop observed in Row (3) is likely due to the extreme class imbalance introduced by real-world Cars, which biases the optimization away from rare open-vocabulary classes.

### 5.6. Visualization

**Figure 8:**
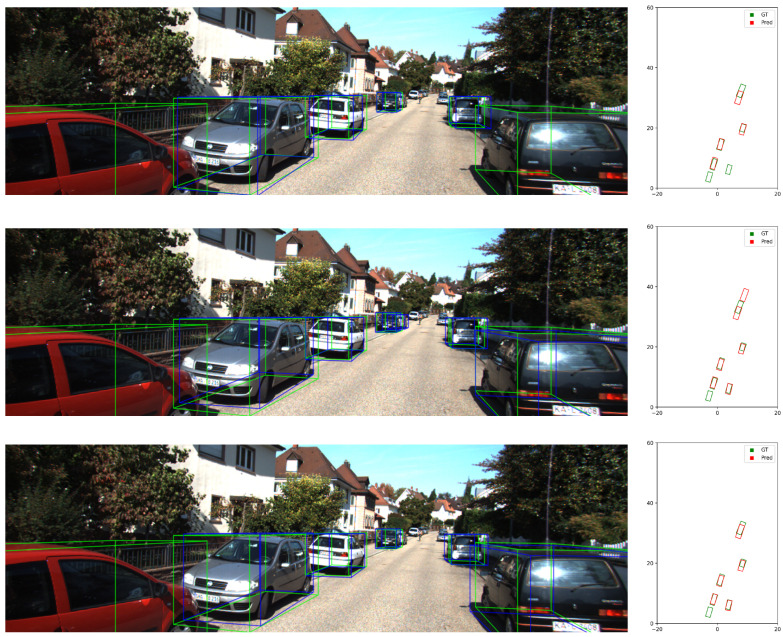
Detection visualization on KITTI. (**Left**): front view with GT (blue) and predictions (red). (**Right**): BEV view with GT (green) and predictions (red).

**Figure 9:**
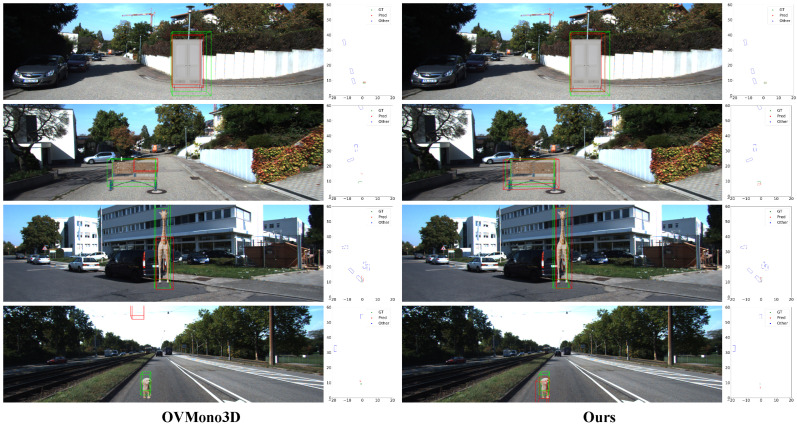
Qualitative comparison on OV-KITTI. Our method improves detection and localization, especially for distant objects.

To provide intuitive insights into the performance of our CLIP-Mono3D model, we present qualitative visualizations of detection results on both the KITTI and OV-KITTI datasets, as shown in Figure 8 and Figure 9.

Figure 8 visualizes detection results on KITTI. Our method exhibits higher recall for distant vehicles compared to MonoDETR. While sharing a similar architecture to MonoDGP, the integration of CLIP-derived semantics improves 2D detection performance, which subsequently leads to more accurate distance regression. These results confirm that semantically enriched 2D features provide robust spatial priors for 3D localization, especially for long-range objects where geometric cues are often ambiguous.

Figure 9 compares CLIP-Mono3D with OVMono3D on the OV-KITTI benchmark. While OVMono3D occasionally yields more precise 2D boxes, our framework demonstrates superior 3D geometric reasoning. Specifically, our depth-guided transformer and language-aware queries enable more accurate depth estimation (row 2, “bench”), enhanced bounding-box integrity for complex shapes (row 3, “giraffe”), and significantly reduced missed detections for small objects (row 4, “dog”), highlighting the robustness of our end-to-end semantic–geometric alignment in open-world scenarios.

### 5.7. Real-World Scenario Verification

To verify generalization in unconstrained real-world environments, we extended our evaluation to the standard KITTI and Argoverse datasets. For KITTI, we adopted a cross-category split by training solely on Car and Pedestrian and evaluating zero-shot performance on Cyclist. For Argoverse, we trained on common categories and evaluated on seven distinct novel classes.

To adapt to the complex nature of the Argoverse dataset, we modified our query initialization strategy. Specifically, we shifted from the global mean aggregation used in OV-KITTI to a discrete spatial assignment paradigm. This ensured that queries were initialized at distinct spatial locations, effectively preventing multiple queries from collapsing onto a single salient object in complex scenes.

As shown in Table 10, on the KITTI dataset, despite the extreme scarcity of training categories limiting the learning of generalized 3D shape priors, our end-to-end framework still achieves competitive performance. It is worth noting that the baseline OVMono3D utilizes an external open-vocabulary 2D detector for strong 2D localization, whereas our method operates seamlessly without explicit 2D bounding-box guidance. On the larger scale Argoverse dataset detailed in Table 11, our method demonstrates robust generalization. Supported by the updated initialization strategy, our model successfully guides geometric reasoning for novel objects, achieving promising results across various categories and obtaining a higher overall average precision compared to the baseline.

**Figure 10:**
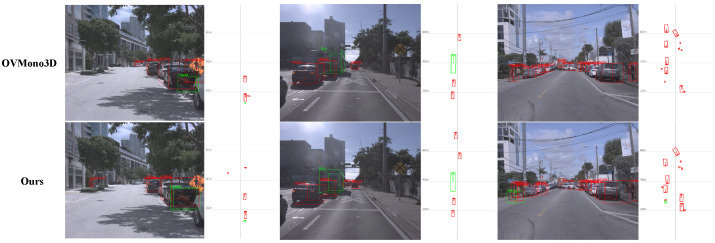
Qualitative comparison on Argoverse. Green boxes represent unseen categories, and red boxes indicate seen categories.

To further illustrate the practical performance, we provide qualitative comparisons in Figure 10. In the first column, our method successfully detects the occluded moped while maintaining the detection completeness of the surrounding objects. In the third column, our model manages to recall the challenging animal class in the distance without missed detections. We acknowledge that the geometric regression for highly irregular novel classes like animals still requires further improvement and exhibits certain deviations. This is primarily due to the unreasonable category distribution inherent in the dataset and the extreme difficulty of these highly crowded scenes.

## 6. Conclusions

We presented CLIP-Mono3D, an end-to-end open-vocabulary monocular 3D detector that directly integrates vision–language semantics without relying on pre-trained 2D detectors. To facilitate comprehensive evaluation, we introduced OV-KITTI, a new benchmark with balanced category distributions. Furthermore, we validated our framework on real-world datasets including KITTI and Argoverse. Extensive experiments demonstrated that our method achieved state-of-the-art performance, delivering a +1.72% $AP_{3D}$ absolute gain on the KITTI easy split and a +1.21% $AP_{15}$ overall improvement on OV-KITTI unseen categories over competitive baselines.

Despite these advances, scaling monocular 3D detection to completely unconstrained open-world environments remains challenging. The primary limitations lie in the inherent scarcity of diverse, real-world 3D annotations and the domain gaps encountered during cross-scene generalization. Achieving true generalized perception requires vastly increasing the scene richness of 3D datasets, maintaining a broad distribution of highly irregular novel classes, and developing targeted adaptation strategies for entirely new domains. Nevertheless, our CLIP-Mono3D provides a viable paradigm to mitigate this data bottleneck by grounding 3D geometry in rich, pre-trained language semantics. Moving forward, future work will focus on alleviating these geometric data constraints, designing robust cross-scene adaptation mechanisms, and extending our semantic–geometric alignment to other data-scarce modalities, such as infrared imaging [51,52,53], to build reliable perception systems under adverse conditions. We hope this work inspires further research in semantic–geometric fusion for 3D perception.

## Figures and Tables

**Table 1:** Comparison of common 3D object detection datasets.

Dataset	Classes	Scene	Modality	Object Range
KITTI 3D	9	Outdoor	RGB, PC	Traffic agents
SUN RGB-D	20	Indoor	RGB-D	Item
ScanNet V2	21	Indoor	RGB-D, PC	Item
Nuscenes	23	Outdoor	RGB, PC	Traffic agents
Objectron	9	Object-wise	RGB	Item
OV-KITTI (ours)	49	Outdoor	RGB	Traffic agents, item, animal

**Table 2:** Training and test set distribution of OV-KITTI.

Category	Class Names	Quantity
Train	Air conditioner, alligator, fireplug, bathtub, bed, volleyball,	5936
	camel, cat, chair, cow, deer, baby buggy, sofa, hippopotamus,	
	horse, lion, oven, pillow, refrigerator, rhinoceros, sculpture,	
	suitcase, telephone booth, television set, wheelchair, zebra,	
	automatic washer, goat, hog, snowman, soccer ball, elephant	
Test	Bench, dog, giraffe, guitar, table, tiger, bucket, wardrobe	1545

**Table 3:** Comparisons with SOTA monocular methods. Best results and our proposed CLIP-Mono3D (“Ours”) are bolded; ⋆: reproduced in our setting. Red and green texts indicate performance gains and drops, respectively.

Method	KITTI (3D)			KITTI (BEV)			OV-KITTI	
	Easy	Mod.	Hard	Easy	Mod.	Hard	AP_25_	AP_50_
MonoDTR [21]	21.99	15.39	12.73	33.33	25.35	21.68	–	–
GUPNet [14]	22.76	16.46	13.72	31.07	22.94	19.75	10.87	2.53
MonoCon [47]	26.33	19.01	15.98	–	–	–	12.15	2.70
MonoCD [48]	26.45	19.37	16.38	34.60	24.96	21.51	12.21	2.74
FD3D [49]	28.22	20.23	17.04	36.98	26.77	23.16	–	–
MonoDETR ⋆ [22]	28.56	19.92	16.17	37.59	26.78	22.79	12.76	3.05
MonoDGP ⋆ [6]	29.68	21.95	18.58	37.87	27.54	24.63	13.33	3.94
**Ours**	**31.40**	**22.98**	**19.61**	**39.47**	**28.72**	**24.93**	**14.44**	**4.88**
Gain	+1.72	+1.03	+1.03	+1.60	+1.18	+0.30	+1.11	+0.94

**Table 4:** Efficiency comparison. ⋆: reproduced in our setting. Note that the parameter count refers to trainable parameters. Best results and our proposed CLIP-Mono3D (“Ours”) are bolded.

Method	#Param (M)	FLOPs (G)	Latency (ms)
MonoDETR ⋆	**39.82**	**47.2**	**33**
MonoDGP ⋆	43.33	55.19	36
**Ours**	43.48	72.52	51

**Table 5:** Open-vocabulary 3D detection performance on OV-KITTI ($AP_{15}$). ^†^ denotes fine-tuning (FT) on the base training set. Best results and our proposed CLIP-Mono3D (“Ours”) are bolded; Red and green texts indicate performance gains and drops, respectively.

Methods	Bench	Table	Tiger	Bucket	Wardrobe	Guitar	Giraffe	Dog	Overall
G-DINO-3D	1.33	2.25	5.48	0.03	7.67	0.00	0.85	0.85	2.31
Gain from FT	+1.63	+2.02	+7.63	+0.64	+1.64	+0.13	+2.44	+0.78	+2.11
G-DINO-3D ^†^	2.96	4.27	13.11	0.67	9.31	0.13	3.29	1.63	4.42
OVMono3D	2.08	3.85	8.96	0.13	8.71	0.04	2.17	0.18	3.27
Gain from FT	+1.19	+0.61	+7.02	+0.49	+2.42	+0.15	+1.61	+2.14	+1.33
OVMono3D ^†^	3.27	4.46	15.98	0.62	**11.13**	0.19	3.78	2.32	4.60
**Ours**	**3.70**	**4.74**	**18.71**	**0.81**	10.70	**0.26**	**4.82**	**2.76**	**5.81**
Gain	+0.43	+0.28	+2.73	+0.19	−0.43	+0.07	+1.04	+0.44	+1.21

**Table 6:** Ablation study on core components and visual–language encoders, highlighting the impact of freezing CLIP parameters; Best results are bolded.

**Method**	**CV**	**OV**
Baseline	13.33	3.94
+Feature Fusion	14.22	4.69
+Query Init.	**14.44**	**5.81**
**Text Encoder **	**CV**	**OV**
CLIP (Frozen)	14.05	5.32
FineCLIP (Frozen)	14.31	5.63
FG-CLIP (Finetuned)	14.15	5.45
FG-CLIP (Frozen)	**14.44**	**5.81**

**Table 7:** Design of language-aware query initialization. NQ represents the number of object queries; Agg. indicates the pooling method (Mean or Max) used to distill global context from keypoints; Best results are bolded.

Config	NQ	Agg.	CV	OV
(a) Learnable	50	-	13.33	3.94
(b) Top-K	50	-	13.95	4.80
(c) +Global	25	Mean	14.28	5.41
(d) +Global	50	Mean	**14.44**	**5.81**
(e) +Global	75	Mean	14.39	5.65
(f) +Global	50	Max	14.09	5.17

**Table 8:** Comparison of cross-modal fusion strategies across different backbone stages and architectural designs.

Fusion	Loc.	CV	OV
(a) None	-	13.33	3.94
(b) Cat	*res2*	13.67	4.18
(c) Add (1L)	*res2*	13.99	4.36
(d) Add (2L)	*res2*	**14.22**	**4.69**
(e) Add (3L)	*res2*	14.14	4.58
(f) Add (2L)	*res3*	13.92	4.41

**Table 9:** Robustness evaluation under real-synthetic data mixing. Seen AP is evaluated exclusively on the original 32 synthetic categories to ensure a fair comparison against the baseline.

Training Data	Seen	Unseen
(1) Pure OV	15.32	5.81
(2) +Ped./Cyc.	15.51	5.76
(3) +Real Car	15.18	4.97

**Table 10:** Zero-shot evaluation on KITTI “Cyclist”. Models were trained only on Car and Pedestrian. Metric: $AP_{15}$ for 3D and BEV for easy and moderate settings. Best results and our proposed CLIP-Mono3D (“Ours”) are bolded.

Method	Cyclist ${\mathrm{AP}}_{3D}$		Cyclist ${\mathrm{AP}}_{\mathrm{BEV}}$	
	Easy	Mod.	Easy	Mod.
OVMono3D	**6.70**	**9.05**	4.35	**5.27**
**Ours**	6.51	7.82	**4.54**	4.99

**Table 11:** Open-vocabulary performance on Argoverse. Evaluated on 7 unseen categories. Best results and our proposed CLIP-Mono3D (“Ours”) are bolded; Red and green texts indicate performance gains and drops, respectively.

Method	Animal	Bus	Emer. Veh.	Moped	Motor.	M.Cyc	Stroller	Overall
OVMono3D	1.56	**23.60**	**4.93**	3.03	14.11	14.49	0.35	8.87
**Ours**	**2.91**	19.88	4.82	**5.96**	**14.37**	**17.12**	**0.72**	**9.40**
Gain	+1.35	−3.72	−0.11	+2.93	+0.26	+2.63	+0.37	+0.53

## Data Availability

Publicly available datasets were analyzed in this study. The KITTI dataset can be found at http://www.cvlibs.net/datasets/kitti/ and the Argoverse dataset at https://www.argoverse.org/. The newly curated OV-KITTI benchmark dataset presented in this study will be publicly available in the repository at: https://github.com/ZC0102-shu/CLIP-Mono3D (all accessed on 10 March 2026).
